# Exploring the Validity of the Velocity Matters Linear Position Transducer in the Back Squat and Bench Press

**DOI:** 10.3390/s26041305

**Published:** 2026-02-18

**Authors:** Emanuele Dello Stritto, Antonio Gramazio, Ruggero Romagnoli, Aristide Guerriero, Claudio Quagliarotti, Maria Francesca Piacentini

**Affiliations:** 1 Department of Human Movement and Health Sciences, University of Rome “Foro Italico”, Piazza L. De Bosis 15, 00135 Rome, Italy; e.dellostritto@studenti.uniroma4.it (E.D.S.); a.gramazio@studenti.uniroma4.it (A.G.); c.quagliarotti@unifortunato.eu (C.Q.); 2 Department of Theoretical and Applied Sciences, eCampus University, 22060 Novedrate, Italy; ruggero.romagnoli@uniecampus.it; 3 Italian Weightlifting Federation, 00196 Rome, Italy; aristide.guerriero@gmail.com; 4 Faculty of Law, Telematic University Giustino Fortunato, 82100 Benevento, Italy

**Keywords:** velocity-based training, training monitoring, encoder, strength training

## Abstract

The purpose of the present study was to validate a new linear encoder by comparing the mean velocity (MV) and peak velocity (PV) of two linear position transducers during free-weight back squat (SQ) and bench press (BP) exercises. Barbell velocity was simultaneously recorded using GymAware (version 5.1.0; reference standard) and Velocity Matters. Fifteen male participants completed two testing sessions, each involving six repetitions (two sets of three) across five velocity ranges: >1.00 to 0.51 m·s^−1^ (velocity range 1: >1.00 m·s^−1^; velocity range 2: 0.87–0.99 m·s^−1^; velocity range 3: 0.75–0.86 m·s^−1^; velocity range 4: 0.63–0.74 m·s^−1^; velocity range 5: 0.51–0.62 m·s^−1^) in SQ and >1.02 to 0.40 m·s^−1^ (velocity range 1: >1.02 m·s^−1^; velocity range 2: 0.86–1.01 m·s^−1^; velocity range 3: 0.70–0.85 m·s^−1^; velocity range 4: 0.56–0.69 m·s^−1^; velocity range 5: 0.40–0.55 m·s^−1^) in BP. In total, 180 repetitions per velocity range were analyzed for each exercise. Validity was assessed using Pearson’s correlation (r), mean absolute error (MAE), Bland–Altman plots, the intraclass correlation coefficient (ICC), and the concordance correlation coefficient (CCC). Pearson’s r indicated good (0.5–0.7) to excellent (>0.9) correlations across all ranges and exercises. However, acceptable MAE values were found only for MV in SQ (except at >1.00 m·s^−1^) and for both MV and PV in BP at velocities <0.70 m·s^−1^. Despite an acceptable MAE in some cases, Bland–Altman analyses revealed systematic underestimation by Velocity Matters, with wide limits of agreement of up to −0.08 m·s^−1^ in SQ and −0.09 m·s^−1^ in BP, even where MAE was acceptable. ICC values were generally >0.70 but showed wide confidence intervals, indicating high uncertainty. CCC values were consistently poor (<0.90) across all velocity ranges and both exercises, except for PV in the lowest velocity range during BP. In conclusion, Velocity Matters may be cautiously used to monitor MV during SQ at velocities <0.87 m·s^−1^, but it does not provide sufficient accuracy for use in BP across any load.

## 1. Introduction

Velocity-based training (VBT) is a method for prescribing and monitoring resistance training sessions that has grown in popularity in recent years [1,2]. Effective implementation requires that the concentric phase of each repetition be executed with maximal intent. Typically, the velocity of the first repetition is used to modulate training intensity [1], while the decrement in velocity within a set serves to regulate training volume [1]. Three velocity metrics are commonly used within the VBT framework [1]: mean velocity (MV), mean propulsive velocity (MPV), and peak velocity (PV). MV represents the average velocity of the barbell throughout the entire concentric phase, measured from the start of the concentric phase until the barbell reaches its maximum height [1]. MPV is defined as the average velocity during the portion of the concentric phase in which the acceleration of the bar remains greater than gravitational acceleration [1]. PV, on the other hand, refers to the maximal instantaneous velocity reached by the barbell during the concentric phase [1]. While PV is commonly used for the monitoring of velocity during ballistic movements [1], MV and MPV are typically preferred for non-ballistic movements [1]. Thus, devices to measure and monitor barbell velocity are essential for an accurate prescription during VBT [3,4].

In this regard, three-dimensional motion capture techniques such as “Vicon” are considered the gold standard for assessing barbell velocity [3]. However, the camera setup for this method is very expensive, as well as impractical for real training settings. Therefore, other devices, such as Linear Position Transducers (LPTs), Linear Velocity Transducers (LVTs), Inertial Measurement Units (IMUs), and smartphone apps, have been widely used as alternatives for prescribing VBT sessions [4,5]. For this reason, the validity and reliability of all these devices have been investigated in order to give important feedback to strength training coaches [5,6,7].

Current research supports the use of LVTs and LPTs as the preferred tools for accurate training prescription [3,4]. Although both LPTs and LVTs use a cable attached to the barbell, they rely on two different methods to determine movement velocity [8,9]. An LVT provides velocity measurements by recording the electrical signals generated proportionally to the cable’s extension velocity [8,9]. In contrast, LPTs calculate movement velocity based on the recorded displacement of the cable [8,9]. Due to their high accuracy and the possibility of providing real-time velocity feedback, GymAware (LPT) and T-Force (LVT) have been used as gold standards to assess the validity and reliability of other devices when 3D motion capture is not available [10,11,12]. In particular, T-force provides more accurate velocity data during exercises performed on the Smith machine [6,9]. However, GymAware appears to be more accurate for free barbell exercises, as it can distinguish between the cable’s horizontal and vertical displacement [6]. Unfortunately, the costs of these two devices remain too expensive (>2000$) [4,13,14] for the majority of strength and conditioning coaches, especially if more than one is needed, such as in team settings. For this reason, recently, a new low-cost LPT named ‘Velocity Matters’ became available on the market at an approximate cost of 300 USD. Despite its growing popularity, no studies have yet assessed its validity. Moreover, the manufacturer does not provide information regarding the device’s technical specifications, such as the sampling frequency, the method used to measure velocity, or whether angular displacement is taken into account. Therefore, the present study aims to verify the validity of Velocity Matters in providing MV and PV metrics during two free barbell exercises: the Bench Press (BP) and Back Squat (SQ). The validity of the device was tested across five velocity ranges for each exercise.

## 2. Materials and Methods

### 2.1. Participants

The present study included a total of 15 well resistance-trained males who regularly trained approximately 3 times per week in a gym, meeting the criteria to be classified as tier 2 according to Mckay et al. [15] (age: 25.4 ± 3.1 years; body mass: 80.7 ± 9.3 kg; height: 1.80 ± 0.05 m; 5.8 ± 2.8 years of experience). Inclusion criteria were: a minimum of two years of resistance training experience, aged between 18 and 35 years, and absence of musculoskeletal injuries in the year preceding the study. Subjects who did not meet all the inclusion criteria or were unable to perform the SQ and BP with proper technique were excluded from the study. Prior to participation, all subjects were fully informed about the procedures, potential risks and ethical considerations of the study and received a detailed information sheet. Written informed consent and authorization for data processing were obtained from each participant. The study was conducted in accordance with the latest revision of the Declaration of Helsinki, which is designed to safeguard the rights, well-being, and dignity of individuals participating in research, and received approval from the appropriate Institutional Review Board (CAR 161/2023).

### 2.2. Data Collection

The GymAware device, considered the gold standard in this study, was used as the reference to verify whether the selected loads fell within the intended velocity range. In total, across the two testing sessions, 900 repetitions per exercise were analyzed, with 180 repetitions recorded for each velocity range. Two different devices were used in the study: GymAware gold standard; (GymAware PowerTool, Kinetic Performance Technologies, Canberra, Australia) and Velocity Matters (Green Wellness, Novara, Italy). The two devices were placed on metal plates on the same side of the barbell, positioned side by side to prevent potential misalignment between devices during data collection. Data recorded during exercise execution were collected by two independent researchers using the proprietary applications of the tested devices. Through these applications, measurements were recorded and exported to Excel (version 2601) and/or SPSS (version 25.0) files for subsequent statistical analyses. As no information is available regarding whether the Velocity Matters device accounts for angular displacement, the devices were positioned as perpendicular as possible to the direction of movement. In line with common practice for linear encoders, the encoder cable was aligned as horizontally as possible relative to the movement to minimize potential measurement error. Finally, we recorded the total number of repetitions per exercise and the velocity range from both devices in order to account for any repetitions that were performed correctly but not registered by the Velocity Matters device.

Participants visited the laboratory on two separate occasions. Two separate sessions were performed because collecting all the data required for the analyses in a single session would have resulted in an excessively long and fatiguing protocol for the participants, potentially compromising the technical quality of exercise execution. During the first session, they provided informed consent and received a detailed information sheet outlining the study procedures and testing protocols. Upon signing of the informed consent documents, participants provided their self-reported one-repetition maximum (1RM) for both the bench press and the back squat. Based on exercise-specific load–velocity relationships defined in the literature [16,17], the load corresponding to the five different velocity ranges was calculated for each participant for both exercises. The velocity ranges were the following: squat: velocity range 1, >1.00 m·s^−1^; ~35–45%1RM; velocity range 2, 0.87–0.99 m·s^−1^; ~45–55%1RM; velocity range 3, 0.75–0.86 m·s^−1^; ~55–65%1RM; velocity range 4, 0.63–0.7 m·s^−1^; ~65–75%1RM; velocity range 5, 0.51–0.62 m·s^−1^; ~75–85%1RM; bench press: velocity range 1, >1.02 m·s^−1^; ~35–45%1RM; velocity range 2, 0.86–1.01 m·s^−1^; ~45–55%1RM; velocity range 3, 0.70–0.85 m·s^−1^; ~55–65%1RM; velocity range 4, 0.56–0.69 m·s^−1^; ~65–75%1RM; velocity range 5, 0.40–0.55 m·s^−1^; ~75–85%1RM. The sets in each session were performed in order of velocity (highest to lowest) for both exercises (from velocity range 1 to velocity range 5). Lighter loads (i.e., <30%RM) were excluded, as they are rarely used during typical training sessions [5], while heavier loads were avoided to prevent excessive fatigue that could impair the following set [5]. During data collection, if the selected load resulted in a movement velocity outside the target velocity range, it was subsequently adjusted and the set was repeated. After 48–72 h following the first session, participants completed the second testing day. The second day followed the same protocol as Day 1, using the same loads previously identified. However, if the velocity initially associated with a given load did not fall within the appropriate velocity range, the load was adjusted to match the participant’s daily condition, as would occur during a training session prescribed using VBT.

### 2.3. Test Procedures

Anthropometric measurements were collected at the beginning of the first session. In both sessions, participants performed a standardized warm-up consisting of five minutes of cycling at a self-selected pace, followed by each participant’s personal mobility routine. In each session, participants performed both the free-weight back squat and the bench press exercises. In the first session, the order of the exercises was randomized and counterbalanced, then reversed for the second session (i.e., if a participant performed the free-weight squat first during session 1, they started with the bench press in session 2 and vice versa). For each exercise, they completed two sets of three repetitions, separated by a three-minute rest, across the five aforementioned velocity ranges. Participants were instructed to perform both the bench press and the squat using their usual technique while executing the concentric phase with maximal intentional velocity during the whole phase. The eccentric phase velocity was not standardized in order to avoid modifications in individual lifting habits and execution patterns. Squat depth was standardized and required to reach the parallel position. In addition, the technical instruction was to include a brief pause between the end of the eccentric phase and the onset of the concentric phase, particularly when lifting lighter loads [18]. Correct technical execution, as described above, was assessed by R.R., a researcher involved in the project and a referee for the Italian Weightlifting Federation (FIPE).

### 2.4. Statistical Analysis

The validity of MV and PV values provided by Velocity Matters was analyzed. The data distribution was tested for normality using the Shapiro–Wilk test, which confirmed normal distribution (*p* > 0.05). Validity was assessed separately for the exercise and velocity range. For the validity analysis, all 180 data points collected per velocity range across the two testing days were analyzed together. Validity was assessed using Bland–Altman plots, mean absolute error (MAE), mean absolute percentage error (MAPE), Pearson’s correlation coefficient (r), the coefficient of determination (R^2^), the intraclass correlation coefficient (ICC; model 3, form 1), and Lin’s concordance correlation coefficient (CCC). The ICC was interpreted as poor when <0.50, moderate between 0.50 and 0.75, good between 0.75 and 0.90, and excellent when >0.90 [19]. The CCC was considered poor when <0.90, moderate between 0.90 and 0.95, substantial between 0.95 and 0.99, and almost perfect when >0.99 [20]. Pearson’s r was interpreted as trivial when <0.10, low between 0.10 and 0.30, moderate between 0.30 and 0.50, high between 0.50 and 0.70, very high between 0.70 and 0.90, and nearly perfect when >0.90. Validity was considered acceptable when MAE was below 0.05 m·s^−1^ (corresponding to ~5%1RM [21,22]), ICC was greater than 0.75, r > 0.70 and CCC was greater than 0.90. Lastly, it was required that the Velocity Matters device did not miss the measurement of any repetition for each velocity range tested in both exercises.

## 3. Results

### 3.1. Free-Weight Back Squat

During the back squat validity assessments, no repetitions were missed in any velocity range. The mean values of MV and PV for each velocity range and device are presented in Table 1. Velocity Matters reported similar MAE and MAPE across the five different velocity ranges and showed greater precision in MV compared to PV (Table 1). Pearson’s correlation coefficient (r) showed values ranging from very high (0.823) to nearly perfect (0.926) in MV (Table 1), while all values in PV fell within the nearly perfect correlation range (>0.90).

**Figure 1:**
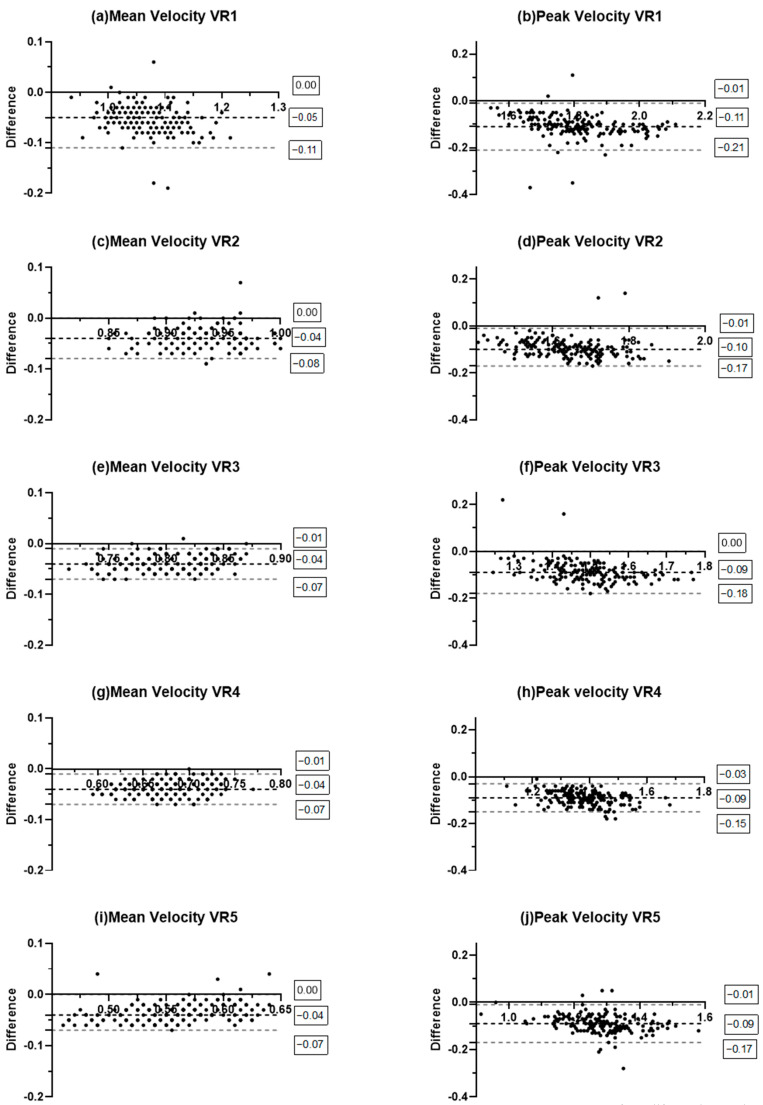
Bland–Altman plot for Velocity Matters and GymAware in free-weight back squat. VR1: velocity range >1.00 m·s^−1^; VR2: velocity range of 0.87–0.99 m·s^−1^; VR3: velocity range of 0.75–0.86 m·s^−1^; VR4: velocity range of 0.63–0.74 m·s^−1^; VR5: velocity range of 0.51–0.62 m·s^−1^.

The Bland–Altman plots indicate that Velocity Matters systematically underestimates MV (Figure 1). A similar underestimation is observed for PV, although with a larger margin of error. Notably, unlike the MV results, the PV analysis shows several data points falling well outside the 95% limits of agreement (LoA), indicating the presence of potential random errors.

The ICC for MV ranged from moderate (0.656) to good (0.818) (Table 2), whereas all ICC values for PV fell within the good range (>0.70) (Table 2). However, notably wide 95% confidence intervals were observed (Table 2). Finally, for the CCC, poor values were reported across all analyzed velocity range, with values of 0.691 or lower for both MV and PV in all velocity ranges (Table 2).

### 3.2. Bench Press Results

During the bench press validity assessments, the Velocity Matters device failed to record 49 out of 900 repetitions, corresponding to 5.4% of the total. Of these 49 unrecorded repetitions, 6 occurred in velocity range 1, 6 in velocity range 2, 13 in velocity range 3, 9 in velocity range 4, and 15 in velocity range 5. The mean values of MV and PV for each velocity range and device are presented in Table 3. Velocity Matters reported a lower MAE in velocity ranges 3 to 5. Moreover, greater precision in MV compared to PV was found only in velocity range 1 and velocity range 2 (Table 3). Pearson’s correlation coefficient (r) showed values ranging from high (0.628) to very high (0.851) in MV (Table 3), while all values in PV fell within the nearly perfect correlation range (>0.90).

The ICC for MV ranged from poor (0.314) to good (0.800) (Table 2), whereas for PV, values ranged from good (0.701) to nearly perfect (0.967) (Table 2). However, notably wide 95% confidence intervals were observed (Table 2). Lastly, CCC indicated poor agreement across all tested loads for both MV and PV (<0.90), except for PV in velocity range 5, which showed a higher value (0.935) (Table 2)

**Figure 2:**
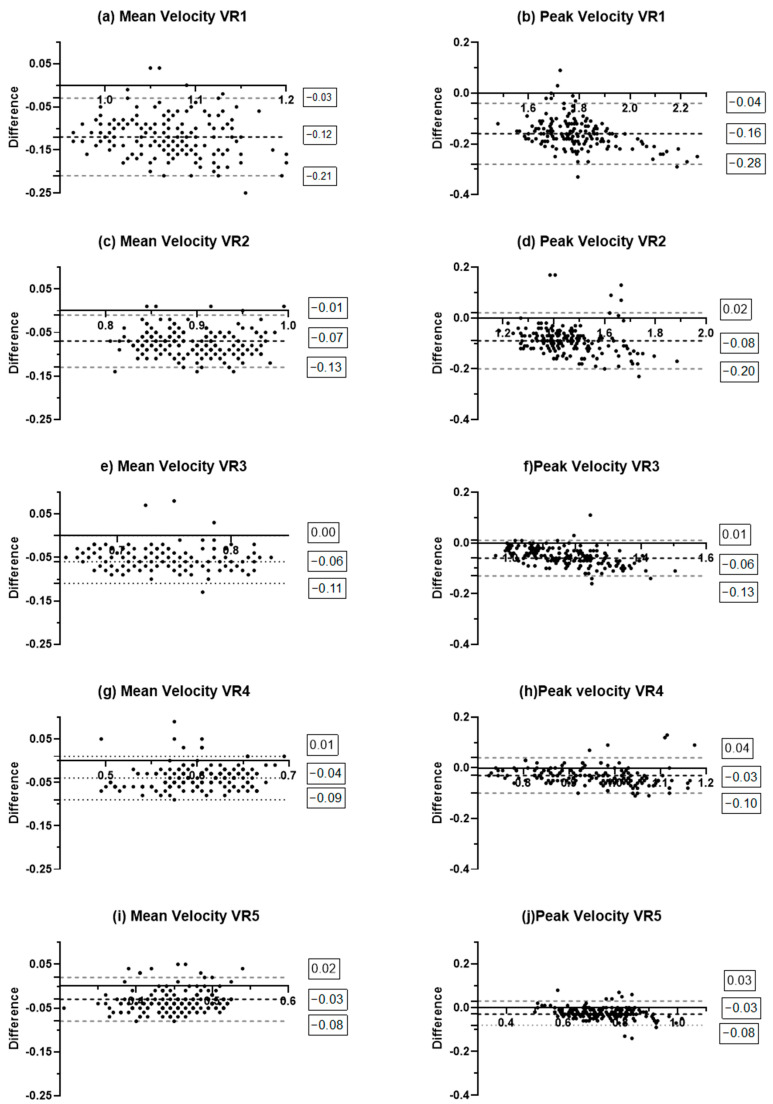
Bland–Altman for Velocity Matters and GymAware in free-weight bench press. VR1: velocity range >1.02 m·s^−1^; VR2: velocity range of 0.86–1.01 m·s^−1^; VR3: velocity range of 0.70–0.85 m·s^−1^; VR4: velocity range of 0.56–0.69 m·s^−1^; VR5: velocity range of 0.40–0.55 m·s^−1^.

The Bland–Altman plots (Figure 2) show that Velocity Matters systematically underestimates MV. A comparable trend is observed for PV, though with a larger margin of error. In contrast to the MV data, the PV analysis displays several points beyond the 95% LoA, suggesting the presence of potential random errors.

## 4. Discussion

The aim of the present study was to assess the validity of Velocity Matters, a new affordable LPT, during two free-weight exercises: SQ and BP. For each exercise, the accuracy of both MV and PV was tested across five velocity ranges spanning from >1.00 m·s^−1^ to 0.51 m·s^−1^ for the SQ and from >1.02 m·s^−1^ to 0.40 m·s^−1^ for the BP. However, since key information regarding the functioning of the Velocity Matters device (e.g., sampling rate, whether angular displacement is accounted for, or the method used to calculate velocity) is not available, it is not possible to make concrete hypotheses regarding the underlying reasons for the observed differences.

### 4.1. Validity of Velocity Matters for Monitoring Back Squat

In the case of SQ, Velocity Matters satisfied the first validity criterion, as it consistently recorded all repetitions without omissions. This could represent an advantage over IMUs, where missed repetitions have previously been reported [4].

Regarding the MV, for all velocity ranges, the analysis revealed a Pearson’s r ranging from high to nearly perfect (Table 1), which, together with good R^2^ values (Table 1), suggests that the measurements of the two devices are highly correlated. Moreover, an acceptable MAE was found for all velocity ranges, except for the lightest one, which exceeded the acceptable error threshold of 0.05 m·s^−1^ (Table 1). However, although the ICC for MV ranged from moderate to good, wide 95%CIs were observed for all velocity ranges (Table 2), which may reflect a high degree of measurement variability, as also evident in the broad LoA (lower limits ranging from −0.07 m·s^−1^ to −0.11 m·s^−1^) in the Bland–Altman analysis (Figure 1). Lastly, poor values of the CCC were found for all velocity ranges, indicating systematic bias between the measurements of Velocity Matters and GymAware (Table 2). These findings may indicate uncertainty in the agreement between the two LPT measurements, suggesting the possibly limited validity of the Velocity Matters.

For PV, Pearson’s r and R^2^ demonstrated nearly perfect correlations between devices (Table 1). However, unacceptable MAE values were found for all velocity ranges (Table 1), suggesting that Velocity Matters is not a valid device for prescribing VBT sessions based on PV. Moreover, the wide 95%CI observed in the ICC, despite good values (Table 2), may indicate high error variability, which is also evidenced by the LoA in the Bland–Altman plots (Figure 1). Moreover, the Bland–Altman analysis showed that Velocity Matters systematically underestimates the PV. Finally, as with MV, poor CCC values for PV were found for all velocity ranges (Table 2).

In summary, for the SQ, Velocity Matters appears to be a suitable device only for the monitoring of MV when target velocities are <0.87 m·s^−1^, as indicated by the MAE (0.04 m·s^−1^) (Table 1). However, due to the wide 95% LoA (lower limits ranging from −0.07 to −0.11) (Figure 1) and the wide 95%CI of the ICC (Table 2), caution should be taken when using Velocity Matters (Green Wellness, Novara, Italy) to monitor MV, regardless of the target velocity.

### 4.2. Validity of Velocity Matters for Monitoring Bench Press

For BP, the first validity criterion was not met by the Velocity Matters device, as it failed to record 49 out of 900 repetitions, corresponding to 5.4% of the total.

Concerning the MV, a high to very high correlation between the two devices was observed, as indicated by Pearson’s r and R^2^ (Table 3). This result, together with the high to nearly perfect correlation found for the SQ, shows that the MV measurements of the Velocity Matters device are correlated with those of the GymAware device, regardless of the exercise performed. Moreover, an acceptable MAE was found for velocity range 4 and velocity range 5, corresponding to the heavier loads (Table 3).

The ICC was also investigated for the MV of this exercise, showing values ranging from poor (velocity ranges 1–3) to good (velocity ranges 4–5) (Table 2). However, the wide ICC 95%CI observed across all velocity ranges (Table 2), together with the wide 95% limit of agreement observed in the Bland–Altman plots for all velocity ranges (Figure 2), may reflect a high degree of measurement variability, suggesting low overall accuracy. Moreover, the Bland–Altman analysis revealed that, as for the SQ, Velocity Matters systematically underestimates MV (lower limits ranging from −0.08 to −0.21) (Figure 2); in addition, for the BP, the presence of random errors was also observed (Figure 2). Lastly, as with the SQ, the CCC observed for MV during the BP was poor across all velocity ranges (Table 2).

Regarding the PV, Pearson’s r, together with R^2^, showed nearly perfect correlation across all velocity ranges (from high to low velocities) between the two LPTs (Table 3), highlighting the consistent pattern of error. Moreover, for velocity ranges 4 and 5, nearly perfect ICC values (Table 2), acceptable 95%CIs (Table 2) and acceptable MAE values (Table 3) were observed. Moreover, for velocity ranges 4 and 5, the CCC also values met the validity criteria (Table 2), as they were considered moderate. For the other velocity ranges (1, 2, and 3) a wide 95%CI and poor CCC were found (Table 2). However, the large dispersion of errors (mainly underestimation) and the presence of random error shown in the Bland–Altman plot (Figure 2) suggest that Velocity Matters is not a fully adequate device for the monitoring of PV, even at slow velocities. However, as previously stated, the validity of PV in the context of non-ballistic exercises remains limited [1].

In summary, for BP, Velocity Matters could be used to monitor PV at slow velocities, considering the acceptable MAE and the compliance with the validity criteria of both the ICC and CCC in velocity ranges 4 and 5. However, caution is needed due to the wide LoA and the large dispersion and random error (Figure 2). Moreover, in the BP, the first validity criterion was not met by Velocity Matters, as 5.4% of the repetitions were missed, which can be considered a limitation of the device. It has to be highlighted that all missed repetitions involved very well-trained athletes who typically perform a slow eccentric phase, regardless of the load. We hypothesize that an excessively slow eccentric velocity may have compromised the detection of repetitions by Velocity Matters, as in all missed repetitions, the eccentric velocity measured by GymAware was below 0.20 m·s^−1^. This could represent a practical limitation of the device, since eccentric velocities below 0.20 m·s^−1^ can often be reached by experienced athletes under submaximal loads, which may reduce the suitability of Velocity Matters for monitoring under these specific conditions. However, the present study failed to systematically investigate this limitation; therefore, further studies are necessary to clarify our hypothesis.

## 5. Conclusions

Velocity Matters failed to meet all validity criteria across the full velocity-range spectrum—both in the BP and in the SQ. Nonetheless, concerning the SQ, measurements for MV appeared to be acceptable for velocities below 0.87 m·s^−1^. However, a constant and substantial underestimation and the variability of MV error must be taken into account. For the PV, the accuracy in the SQ was found to be unacceptable. With regard to the BP, Velocity Matters showed good consistency of measurements and acceptable MAE values only for PV in velocity ranges 4 and 5. However, the occurrence of missed repetitions, probably due to the excessively slow eccentric velocity, along with the wide dispersion of error and the presence of random error, suggests that Velocity Matters may not be considered a valid tool for prescribing VBT sessions in BP exercise during resistance training sessions.

## Figures and Tables

**Table 1:** Differences between GymAware and Velocity Matters in free-weight back squat. VR1: velocity range >1.00 m·s^−1^; VR2: velocity range of 0.87–0.99 m·s^−1^; VR3: velocity range of 0.75–0.86 m·s^−1^; VR4: velocity range of 0.63–0.74 m·s^−1^; VR5: velocity range of 0.51–0.62 m·s^−1^; MAE: mean absolute error; MAPE: mean absolute percentage error.

Mean Velocity						
	GymAware (m·s^−1^)	Velocity Matters (m·s^−1^)	MAE (Mean ± SD)	MAPE (Mean ± SD)	Pearson’s r	Coefficient of Determination (R^2^2)
VR1	1.09 ± 0.06	1.04 ± 0.05	0.06 ± 0.03	5.0 ± 2.3%	0.871	0.76
VR2	0.95 ± 0.03	0.91 ± 0.04	0.04 ± 0.02	4.3 ± 1.9%	0.823	0.68
VR3	0.82 ± 0.03	0.78 ± 0.04	0.04 ± 0.02	4.6 ±1.9%	0.901	0.81
VR4	0.69 ± 0.04	0.66 ± 0.04	0.04 ± 0.01	5.4 ± 2.1%	0.926	0.86
VR5	0.57 ± 0.04	0.54 ± 0.05	0.04 ± 0.02	6.5 ± 2.8%	0.924	0.85
Peak Velocity						
	GymAware (m·s^−1^)	Velocity Matters (m·s^−1^)	MAE (Mean ± SD)	MAPE (Mean ± SD)	Pearson’s r	Coefficient of Determination (R^2^2)
VR1	1.86 ± 0.13	1.76 ± 0.12	0.11 ± 0.05	5.8 ± 2.3%	0.925	0.85
VR2	1.69 ± 0.10	1.59 ± 0.10	0.10 ± 0.03	5.8 ± 1.7%	0.926	0.86
VR3	1.53 ± 0.11	1.45 ± 0.09	0.09 ± 0.04	6.0 ± 2.4%	0.907	0.82
VR4	1.43 ± 0.10	1.33 ± 0.09	0.09 ± 0.03	6.5 ± 2.0%	0.957	0.91
VR5	1.33 ± 0.10	1.24 ± 0.10	0.09 ± 0.03	6.9 ± 2.5%	0.932	0.87

**Table 2:** Intraclass correlation and concordance correlation coefficient results. ICC: intraclass correlation; CCC: concordance correlation coefficient; CI: confidence interval.

Mean Velocity				
	Back Squat		Bench Press	
	ICC (95%CI)	CCC (95%CI)	ICC (95%CI)	CCC (95%CI)
VR1	0.727(−0.183–0.916)	0.570 (0.501–0.632)	0.314 (−0.131–0.665)	0.185 (0.138–0.230)
VR2	0.656 (−0.187–0.887)	0.486 (0.414–0.552)	0.507 (−0.139–0.819)	0.338 (0.276–0.396)
VR3	0.731 (−0.144–0.922)	0.575 (0.509–0.634)	0.641 (−0.173–0.883)	0.470 (0.399–0.535)
VR4	0.760 (−0.120–0.934)	0.611 (0.549–0.667)	0.750 (−0.165–0.916)	0.599 (0.525–0.663)
VR5	0.818 (−0.155–0.949)	0.691 (0.632–0.742)	0.800 (−0.083–0.933)	0.665 (0.595–0.726)
Peak Velocity				
	Back Squat		Bench Press	
	ICC (95%CI)	CCC (95%CI)	ICC (95%CI)	CCC (95%CI)
VR1	0.802 (−0.149–0.945)	0.668 (0.606–0.721)	0.701 (−0.115–0.914)	0.538 (0.475–0.596)
VR2	0.780 (−0.133–0.940)	0.638 (0.577–0.692)	0.850 (−0.100–0.955)	0.738 (0.682–0.785)
VR3	0.786 (−0.169–0.938)	0.640 (0.576–0.695)	0.917 (0.046–0.976)	0.846 (0.809–0.876)
VR4	0.793 (−0.092–0.947)	0.656 (0.599–0.706)	0.944 (0.704–0.978)	0.893 (0.862–0.917)
VR5	0.803 (−0.137–0.947)	0.669 (0.610–0.721)	0.967 (0.823–0.987)	0.935 (0.915–0.950)

**Table 3:** Differences between GymAware and Velocity Matters in free-weight bench press. VR1: velocity range >1.02 m·s^−1^; VR2: velocity range of 0.86–1.01 m·s^−1^; VR3: velocity range of 0.70–0.85 m·s^−1^; VR4: velocity range of 0.56–0.69 m·s^−1^; VR5: velocity range of 0.40–0.55 m·s^−1^; MAE: mean absolute error; MAPE: mean absolute percentage error.

Mean Velocity						
	GymAware (m·s^−1^)	Velocity Matters (m·s^−1^)	MAE (Mean ± SD)	MAPE (Mean ± SD)	Pearson’s r	Coefficient of Determination (R^2^)
VR1	1.12 ± 0.05	1.01 ± 0.05	0.12 ± 0.04	10.5 ± 3.7%	0.628	0.39
VR2	0.93 ± 0.05	0.86 ± 0.05	0.08 ± 0.03	8.0 ± 3.0%	0.785	0.62
VR3	0.77 ±0.05	0.71 ± 0.04	0.06 ± 0.02	7.4 ± 2.6%	0.831	0.69
VR4	0.61 ± 0.04	0.57 ± 0.05	0.04 ± 0.02	7.1 ± 3.2%	0.826	0.68
VR5	0.47 ± 0.04	0.43 ± 0.05	0.04 ± 0.02	8.3 ± 3.9%	0.851	0.72
Peak Velocity						
	GymAware (m·s^−1^)	Velocity Matters (m·s^−1^)	MAE (Mean ± SD)	MAPE (Mean ± SD)	Pearson’s r	Coefficient of determination (R^2^)
VR1	1.87 ± 0.15	1.71 ± 0.13	0.16 ± 0.05	8.6 ± 2.7%	0.920	0.85
VR2	1.50 ± 0.13	1.41 ± 0.12	0.10 ± 0.04	6.3 ± 2.6%	0.911	0.83
VR3	1.20 ± 0.12	1.14 ± 0.10	0.06 ± 0.03	4.6 ± 2.4%	0.961	0.93
VR4	0.97 ± 0.10	0.93 ± 0.10	0.04 ± 0.03	4.2 ± 2.5%	0.941	0.89
VR5	0.75 ± 0.10	0.72 ± 0.10	0.03 ± 0.02	4.0 ± 2.8%	0.966	0.93

## Data Availability

Data available upon request due to restrictions (privacy).

## Abbreviations

The following abbreviations are used in this manuscript:

VBT	Velocity-based training
MV	Mean Velocity
MPV	Mean Propulsive Velocity
PV	Peak Velocity
LVT	Linear Velocity Transducer
LPT	Linear Position Transducer
IMU	Inertial Measurement Unit
BP	Bench Press
SQ	Back Squat
1RM	One-Repetition Maximum
MAE	Mean Absolute Error
MAPE	Mean Absolute Percentage Error
ICC	Intraclass Coefficient Correlation
CCC	Lin’s Concordance Correlation Coefficient
VR	Velocity Range
CI	Confidence Interval
LoA	Limits of Agreement
